# *AceDRG*: a stereochemical description generator for ligands

**DOI:** 10.1107/S2059798317000067

**Published:** 2017-02-01

**Authors:** Fei Long, Robert A. Nicholls, Paul Emsley, Saulius Gražulis, Andrius Merkys, Antanas Vaitkus, Garib N. Murshudov

**Affiliations:** a Structural Studies, MRC Laboratory of Molecular Biology, Francis Crick Avenue, Cambridge CB2 0QH, England; b Institute of Biotechnology, Saulėtekio al. 7, LT-10257 Vilnius, Lithuania

**Keywords:** *AceDRG*, refinement, ligand chemistry, Crystallography Open Database, *RDKit*

## Abstract

The program *AceDRG* is designed for the derivation of stereochemical information about small molecules. It uses local chemical and topological environment-based atom typing to derive and organize bond lengths and angles from a small-molecule database: the Crystallography Open Database (COD). Information about the hybridization states of atoms, whether they belong to small rings (up to seven-membered rings), ring aromaticity and nearest-neighbour information is encoded in the atom types. All atoms from the COD have been classified according to the generated atom types. All bonds and angles have also been classified according to the atom types and, in a certain sense, bond types. Derived data are tabulated in a machine-readable form that is freely available from *CCP*4. *AceDRG* can also generate stereochemical information, provided that the basic bonding pattern of a ligand is known. The basic bonding pattern is perceived from one of the computational chemistry file formats, including SMILES, mmCIF, SDF MOL and SYBYL MOL2 files. Using the bonding chemistry, atom types, and bond and angle tables generated from the COD, *AceDRG* derives the ‘ideal’ bond lengths, angles, plane groups, aromatic rings and chirality information, and writes them to an mmCIF file that can be used by the refinement program *REFMAC*5 and the model-building program *Coot*. Other refinement and model-building programs such as *PHENIX* and *BUSTER* can also use these files. *AceDRG* also generates one or more coordinate sets corresponding to the most favourable conformation(s) of a given ligand. *AceDRG* employs *RDKit* for chemistry perception and for initial conformation generation, as well as for the interpretation of SMILES strings, SDF MOL and SYBYL MOL2 files.

## Abstract 2

The program *AceDRG* generates accurate stereochemical descriptions, and one or more conformations, of a given ligand. The program also analyses entries and extracts local environment-dependent atom types, bonds and angles from the Crystallography Open Database.

## 1. Introduction   

Macromolecular crystallography (MX) is the most widely used experimental technique in structural biology that allows the study of three-dimensional structures of macromolecules in atomic, and sometimes electronic, detail, which is an essential step in understanding biological processes. In recent years, single-particle cryo-EM has made substantial advances (Kühlbrandt, 2014 ▸) and thus is now being used routinely. Both techniques allow the derivation of snapshots of reactions or molecular binding processes. For this type of study, a structure of a single molecule is often not sufficient; it is more common to study structures of macromolecules in complex with small ligands mimicking intermediate states or close to a transition state. Moreover, the quality and quantity of the experimental data are often deficient (low resolution with small signal-to-noise ratio). This means that the data alone are not sufficient to derive chemically and structurally sensible atomic models; the data must be supplemented by prior knowledge pertaining to the chemistry and structure of the molecules under study in order to address the problem of missing high-resolution information (Murshudov *et al.*, 2011 ▸; Nicholls *et al.*, 2012 ▸; Schröder *et al.*, 2010 ▸; Adams *et al.*, 2010 ▸; Smart *et al.*, 2012 ▸). Experimental data produced by MX and cryo-EM usually contain long-range information. As the resolution of the data increases, shorter and shorter-range information becomes available. Owing to the mobility of atoms and dynamic/static disorder, even at very high resolution electronic details are not visible, the signal is reduced and thus local resolution is reduced. Additional information is almost always needed. The most widely used information is that regarding the chemistry of bonds and angles (Vagin *et al.*, 2004 ▸). This was recognized a long time ago, and has been used to stabilize atomic structure refinement when only limited and noisy data are available. For amino acids and nucleic acids the ‘ideal’ values have been tabulated a number of times (Engh & Huber, 1991 ▸, 2001 ▸; Parkinson *et al.*, 1996 ▸). There are several good software tools designed for the derivation of accurate values for the bonds and angles in small molecules (Moriarty *et al.*, 2009 ▸; Smart *et al.*, 2011 ▸; Schüttelkopf & van Aalten, 2004 ▸). These are either based on molecular-mechanics force fields, *Mogul* (Bruno *et al.*, 2004 ▸) from the Cambridge Structural Database (CSD) or semi-empirical quantum-chemical (QM) calculations (Rocha *et al.*, 2006 ▸). Programs such as *LIBCHECK* (Vagin *et al.*, 2004 ▸) and *JLigand* (Lebedev *et al.*, 2012 ▸) available from *CCP*4 (Winn *et al.*, 2011 ▸) can also be used to generate ligand descriptions with sufficient quality.

Information regarding the local geometry of a small compound can be derived using two different approaches.

(i) High-level QM calculations (for example, see Szabo & Ostlund, 1989 ▸). However, this usually takes a long time, and the results are dependent on the description of the environment included in the calculations. Calculations carried out in a vacuum would not represent reality at all; for example, a carboxyl group would always be protonated in a vacuum. In reality, protonation state and hence the geometric details of ligands depend on their immediate environment. In many cases, it is safe to assume that ligands are in an environment with a pH of around 7.0. If QM calculations are used then this assumption must be included in the calculations, for example by adding implicit or explicit solvent.
(ii) High-resolution structures from small-molecule databases, such as the CSD (Groom *et al.*, 2016 ▸) and the Crystallography Open Database (COD; Gražulis *et al.*, 2009 ▸), are a rich source of prior chemical information. Whilst there are around one million structures in the CSD, it is unlikely that the compounds needed for drug design or protein-function inhibition will be present in this source. The purpose of small-molecule studies is very different from that of structural biology. However, it can be expected that the local information will be more or less similar for ligands in the COD/CSD as in those used in structural biological studies.

Although the number of structures (around 367 000) in the COD (Gražulis *et al.*, 2012 ▸) is almost three times lower than that (around 900 000) in the CSD (Groom *et al.*, 2016 ▸), one main advantage of the COD is that it is free, in the sense that all its data have been placed in the public domain by the COD contributors, and derived data can be freely distributed. Therefore, testing developed algorithms using the COD is relatively easy. However, the developed algorithm and its implementation in *AceDRG* is such that any source of reliable data, including the CSD or high-level QM-derived structures, can be used to regenerate/supplement the existing database of atom types, bonds and angles. Moreover, the CSD already offers very good state-of-the-art tools for the derivation of ligand descriptions based on entries in the CSD, specifically *Mogul* (Bruno *et al.*, 2004 ▸). It should be noted that *phenix.elbow* (Moriarty *et al.*, 2009 ▸) from the *PHENIX* software suite (Adams *et al.*, 2010 ▸), *grade* (Smart *et al.*, 2011 ▸) from Global Phasing and *pyrogen* from *Coot* (Emsley *et al.*, 2010 ▸) use *Mogul* to generate accurate ligand descriptions. We decided to develop an alternative algorithm to derive bonds and angles from the COD and generate ligand descriptions. In designing the algorithms and software, we were mindful that the database should be dynamically extensible, *i.e.* as the number of small molecules increases, or new sources of small-molecule structures become available, this database can be updated with little effort.

The Protein Data Bank (PDB; Berman *et al.*, 2002 ▸) is a rich source of information about structures and macromolecular chemistry. The wider community of biologists often use the entries deposited in the PDB without having much background in structural biology. Therefore, it is necessary to make sure that the entries deposited are of sufficient reliability and accuracy, and that they are consistent with the experimental data as well as with prior chemical and structural information. The PDB has done excellent work in the organization of data, including that pertaining to ligand chemistry (Dimitropoulos *et al.*, 2006 ▸; Feng *et al.*, 2004 ▸). However, despite the efforts by the wwPDB, there are still a number of errors in the PDB, especially regarding ligands (Pozharski *et al.*, 2013 ▸; Weichenberger *et al.*, 2013 ▸). There have even been some claims that most of the errors in the PDB are owing to errors in ligands (Liebeschuetz *et al.*, 2012 ▸; Reynolds, 2014 ▸). Most of the errors can be attributed to overinterpretation and misinterpretation of the electron density, with the experimenter having a strong desire to see ligand electron density, which is often the focus of studies involving ligand–protein complexes. However, the number of errors owing to the inaccurate chemical description of ligands is not negligible. In general, it would be very hard to describe ligand geometry if incorrect ligand chemistry is assumed. However, it is possible to reduce such errors by accurately designing a software program with some chemical intelligence. *AceDRG* is designed to reduce such errors, giving a sufficiently accurate ligand description and thus helping to reduce the number of errors in the PDB.

The current version of *AceDRG* makes extensive use of tools available in the computational chemistry toolkit *RDKit* (http://www.rdkit.org).

*Organization of this paper*. In §[Sec sec2]2, we briefly introduce the program *AceDRG*. We then describe atom types including hybridization, ring and aromaticity perception in §[Sec sec3]3. In §[Sec sec4]4, we describe the organization of derived atom-type, bond and angle tables. In §[Sec sec5]5, we describe the derivation of stereochemical information about ligands. §[Sec sec6]6 gives several examples of application. Finally, we summarize the current state and give our views on future perspectives. This paper attempts to describe the algorithms implemented in the program *AceDRG*. The source code is available from *CCP*4 (Winn *et al.*, 2011 ▸) under the LGPL license; further details can be found in the code and documentation.

## 2. *AceDRG*   

*AceDRG* is a multifunctional software tool that analyses molecules in small-molecule databases (currently only the COD), extracts all atom types, bond lengths and angles from those databases, and organizes them in a hierarchical manner. It reads an input file containing basic chemical information about a ligand, such as a bonding graph and stereochemistry. It derives atom types from the bonding graph and maps them to those extracted from the small-molecule database. It can also generate one or more coordinate sets corresponding to energetically favourable conformation(s) of ligands.

## 3. Atom types   

The atom typing used in *AceDRG* encapsulates the local topological and chemical environments of atoms. This includes the atom’s number of bonds and those of its neighbours (up to the third neighbours) and, if they belong to ring(s), information regarding ring size and aromaticity. The current algorithm only considers the extended organic set of atoms: B, C, N, O, S, P, Se, F, Cl, Br, I and H. These atoms cover 93% of the chemical entities contained in the PDB. Dealing with metals requires a different approach; they will be dealt with in the future.

Since the hybridization state of atoms and the number, size and aromaticity of rings play essential roles in atom-type definitions, we shall describe them next.

### 3.1. Hybridization   

Hybridization perception for atoms is performed in several steps. In the first step, for each atom the default hybridization state is assigned using the rules described in Table 1 ▸. In brief, hybridization is defined by the difference between the atomic valence and the number of connections. For example, a C atom with four bonds is always *sp* ^3^, one with three bonds is *sp* ^2^ and one with two bonds is *sp* ^1^ (*sp*). By default, N and B are *sp* ^3^ if they have three or four bonds, and *sp* ^2^ if they have two bonds. Formal charges are also assigned during default hybridization assignment. For example, if N has four bonds then its formal charge is +1 and if B has four bonds then its formal charge is −1 (*i.e.* the difference between the valence and the number of connections). See Table 1 ▸ for more details.

For C atoms the default rules are sufficient. However, for some other atoms, such as N, B and O, further hybridization refinement is needed.

If an atom is N or B and it has three connections then its hybridization state is revised according to the local chemical environment. If accurate atomic coordinates are available for a particular molecule, for example those from the COD after validation (Long *et al.*, 2017 ▸), then we refine the hybridization state as follows. If three atoms are connected to the target atom then three vectors are formed. If all three vectors are co-planar (*i.e.* they are on the same plane) then the target atom is considered to be *sp* ^2^. Co-planarity of vectors is equivalent to the statement that one of the vectors is perpendicular to the normal of a plane formed by the remaining two vectors. If abs(θ − 90°) < *c* then two vectors are considered perpendicular, where θ is the angle between the third vector and the normal of the plane formed by the two remaining vectors. The current value of the parameter *c* is 5°, although it can be readjusted if necessary. This approach was found to be useful when classifying all atoms from the COD. When working with only a bonding graph, such as from a SMILES string (Weininger, 1988 ▸; Weininger *et al.*, 1989 ▸), then this approach is not applicable.

If there are no reliable coordinates available then the decision regarding hybridization state is made according to the local environment of the atom (this part of the algorithm applies for N and B with three bonds).

**Figure 1:**
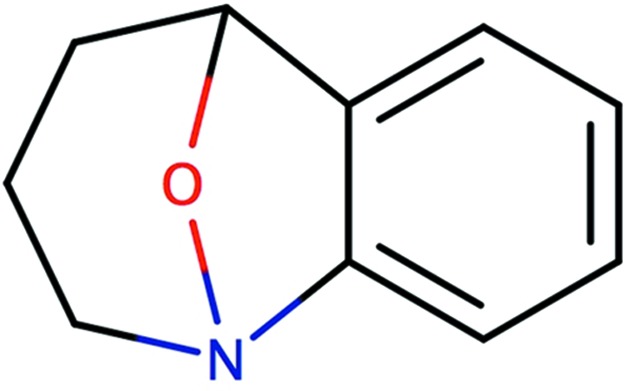
A caged structure where the N atom has hybridization state *sp* ^3^. This figure was produced by *Marvin Sketch* v.16.9.12 (http://www.chemaxon.com).

(i) If the atom is in a caged bridge (Fig. 1 ▸) then it is kept in the *sp* ^3^ state.
(ii) If the atom is connected to another atom that is in an aromatic ring, then we consider it to be *sp* ^2^. It is likely that lone pairs of N will try to align with π electrons of the aromatic system.
(iii) If the atom is connected to an atom that is defaulted to be in the *sp* ^2^ state, then its hybridization state is changed to *sp* ^2^.

The hybridization state of O atom is also refined further. If an O atom with two bonds is connected to at least one H atom then it is considered to be *sp* ^3^. If both connected atoms are not H atoms, and at least one of them has default *sp* ^2^ hybridization, then the O atom is considered to be *sp* ^2^.

The hybridization states of H and halogen atoms are always set to ‘none’, as they do not affect the atom types of connected atoms and therefore their classification.

### 3.2. Ring perception   

The set of smallest rings is determined using a modified version of the algorithm described by Downs *et al.* (1989 ▸). The articles by Figueras (1996 ▸), Hanser *et al.* (1996 ▸) and Leach *et al.* (1990 ▸) were also consulted. Since the rings are used as part of atomic classification in *AceDRG*, we need only obtain information regarding any rings containing the atom being classified. Moreover, we need only consider rings with limited size; in the current version we only use rings containing up to seven atoms. The algorithm can be considered as a limited depth-first search algorithm. The depth of the search depends on the maximum ring size to include. However, the algorithm is flexible enough to be extended to larger ring sizes. The algorithm is as follows.

(i) Loop over all atoms of the compound. At this stage, we select only atoms that are in the extended organic set.
(ii) We refer to the selected atom as the ‘original atom’. We search all neighbours of this atom depth-by-depth. The depth is limited by the maximum ring size to be detected. As soon as the required depth has been reached, the search is stopped.
(iii) We set the selected atom to the current atom.
(iv) We then check all neighbours of the current atom.
(v) If the neighbour is not the ‘original atom’ and it is not the atom that preceded the current atom, and it is in the list of atoms that we have already seen, then we call this neighbour Nachbarpunkt (this terminology is taken from Downs *et al.*, 1989 ▸) and we stop walking further.
(vi) If no Nachbarpunkt is found then we check the current atom’s neighbours one by one to see if one of them closes the ring, *i.e.* if a neighbour atom is the original atom we are classifying. If a ring is closed then we record the ring, together with the information about the atoms contained within this ring. In addition, a Nachbarpunkt is detected because the original atom is always Nachbarpunkt, and thus we stop walking further.
(vii) If no Nachbarpunkt is found after step (vi), we increase the level and check whether the maximum depth has been reached. If not then we put the current atom in the list of ‘seen atoms’ and set the current atom to be the previous atom. Loop over all neighbours of the current atom. For each neighbour atom, recursively go back to step (iii) and set it to be the new current atom.

This algorithm finds all small cycles up to a given size. Moreover, the algorithm also gives the list of atoms belonging to the same ring.

### 3.3. Aromaticity   

A ring or fused-ring system is considered to be aromatic if all atoms belonging to the system are in the *sp* ^2^ hybridization state and the number of π electrons obeys Hückel’s 4*n* + 2 rule (Coulson *et al.*, 1978 ▸), where *n* is an integer. Table 2 ▸ describes the number of π electrons that each atom contributes to the total count. The algorithm and the table are extended versions of those described by Balaban (1985 ▸). In addition to the rules described by Balaban (1985 ▸), we add several rules for S, P and O atoms (Wilberg *et al.*, 2001 ▸; Chivers, 2005 ▸; Chivers & Manners, 2009 ▸; Krygowski *et al.*, 2009 ▸; Fowler *et al.*, 2004 ▸). The whole fused-ring systems are considered first. If the whole system obeys Hückel’s rule then the whole system, and thus each ring in the system, is considered to be aromatic, even though some of the contributing rings may not obey this rule. If the whole system does not obey the 4*n* + 2 rule then each of the smallest rings in the system are considered one by one. If any of the smallest rings obey this rule then it is considered to be aromatic.

#### 3.3.1. Example: FAD and FDA   

**Figure 2:**
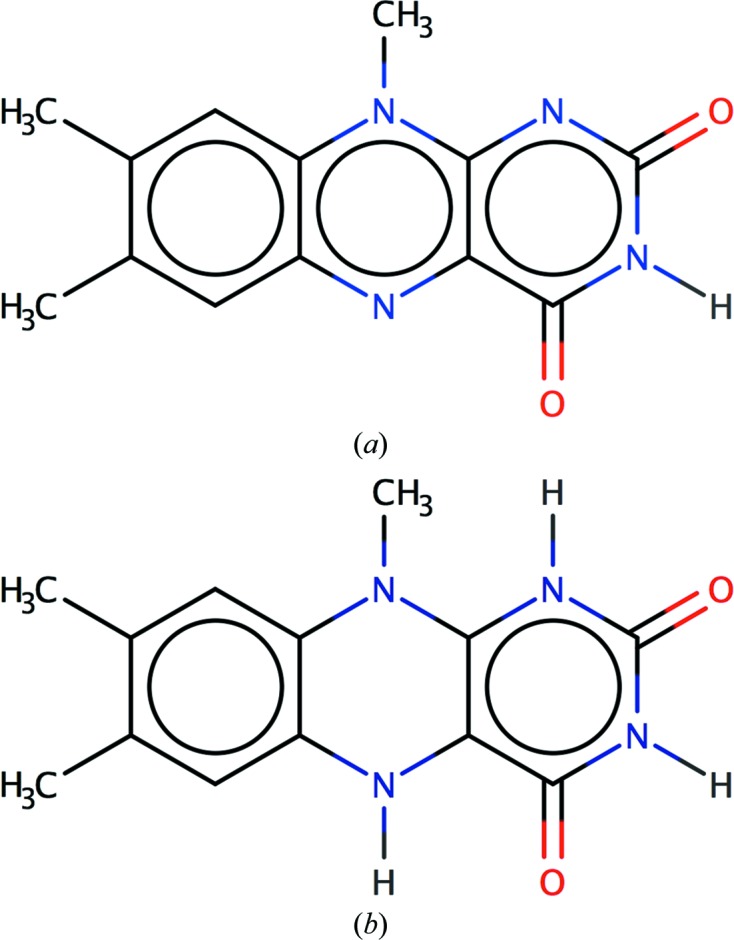
Aromaticity in (*a*) flavin adenine dinucleotide (FAD) and (*b*) dihydroflavine-adenine dinucleotide (FDA). The number of π electrons for (*a*) is 18 and that for (*b*) is 20. In (*b*) the outer rings have six and ten π electrons, respectively. (*a*) is perceived as an aromatic system, whereas in (*b*) only the outer rings are aromatic. This figure was produced by *Marvin Sketch* v.16.9.12 (http://www.chemaxon.com).

Flavin adenine dinucleotide (FAD) is a redox cofactor, and in many biological processes it is converted to dihydroflavine-adenine dinucleotide (FDA) by accepting two electrons and two protons (Fig. 2 ▸). Both FAD and FDA contain fused three-ring systems: flavin groups in oxidized (FAD) and reduced (FDA) forms. However, these fused systems are very different. In FAD all three rings are contained in one aromatic system. In FDA the outer rings are aromatic, whilst the middle ring is not. As a result, the flavin ring plane can be bent more in FDA than in FAD (Walsh & Miller, 2003 ▸).

According to the π-electron count in the flavin of FAD there are 14 π electrons, making it aromatic (14 = 4*n* + 2, with *n* = 3). In FDA the number of π electrons in the ring system is 16 (16 = 4*n* with *n* = 4). Therefore, the flavin of FDA is considered to be anti-aromatic. In FDA the outer rings both contain six π electrons, making both of them aromatic rings, whilst the middle ring contains eight π electrons and thus is not considered to be aromatic.

**Figure 3:**
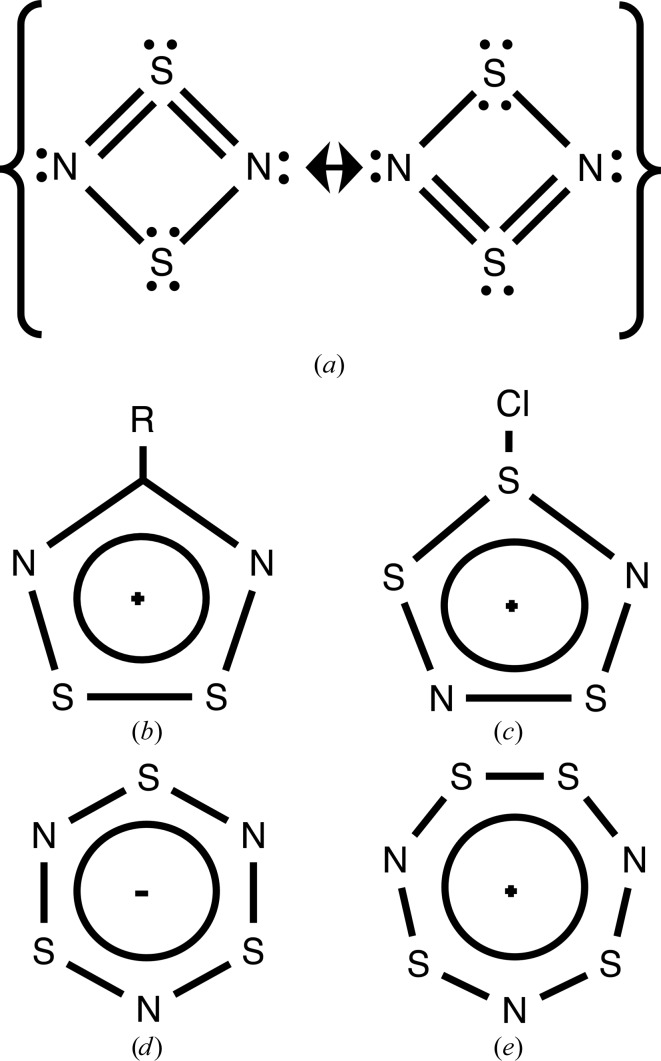
Examples of counting π electrons in sulfur–nitrogen rings where all atoms are *sp* ^2^-hybridized. The total numbers of π electrons are (*a*) six (Wilberg *et al.*, 2001 ▸), (*b*) six (Chivers & Manners, 2009 ▸), (*c*) six (Chivers & Manners, 2009 ▸), (*d*) ten (Chivers & Manners, 2009 ▸), (*e*) eight (Chivers, 2005 ▸), (*f*) ten (Chivers, 2005 ▸) and (*g*) ten (Chivers, 2005 ▸). All rings apart from (*e*) obey Hückel’s 4*n* + 2 rule, where *n* is an integer. Therefore, all rings apart from that in (*e*) are perceived as aromatic rings.

Fig. 3 ▸ shows different numbers of π electrons in a series of sulfur–nitrogen rings, according to the rules shown in Table 2 ▸. These are in agreement with suggestions made in other studies (Wilberg *et al.*, 2001 ▸; Fowler *et al.*, 2004 ▸; Chivers, 2005 ▸; Chivers & Manners, 2009 ▸).

### 3.4. Atom types   

**Figure 4:**
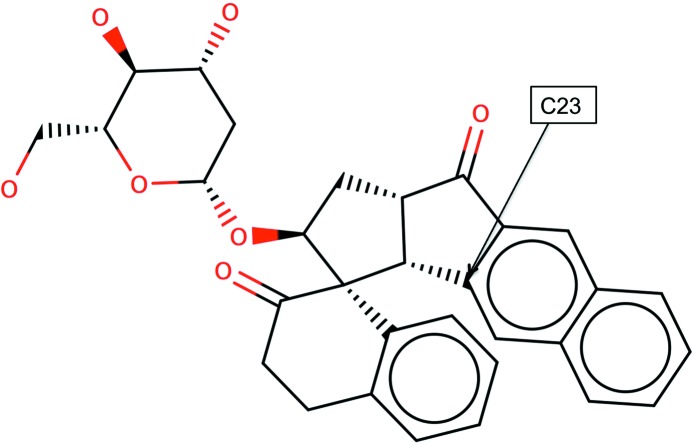
An example of local topology and chemistry-based atom types. This example corresponds to the ligand DDI from the PDB’s CCD. The full atom type of C23 is C[5,6a](C[5,5]C[5,5]C[5,6]H)(C[5,6a]C[6a]C[5])(C[6a]C[6a,6a]H){1|O<1>,2|C<4>,2|H<1>,4|C<3>}. This figure was produced using *Marvin Sketch* v.16.9.12 (http://www.chemaxon.com).

Once the bonding graph, atom hybridization, ring membership, size and aromaticities are known then the atoms can be classified using their local topological and chemical environments. For example, in Fig. 4 ▸ atom C23 is in the class with identifier code C[5,6a](C[5,5]C[5,5]C[5,6]H)(C[5,6a]C[6a]C[5])(C[6a]C[6a,6a]H){1|O<1>,2|C<4>,2|H<1>,4|C<3>}.

This means that the original atom is a C atom and it belongs to a five-membered non-aromatic and a six-membered aromatic ring (represented by C[5,6a]). It has three first neighbours. The first of those neighbours is a C atom, which belongs to two five-membered rings. This neighbour has three second neighbours: one of them is a C atom belonging to two five-membered rings, the next is also a C atom belonging to five and six-membered rings, and the third is an H atom. Obviously, this first-neighbour atom also connects to the original atom. Similarly, the second first neighbour of the original atom is a C atom belonging to two rings: one five-membered non-aromatic ring and one six-membered aromatic ring. This atom also has two additional neighbours: a C atom belonging to a five-membered non-aromatic ring and a C atom belonging to a six-membered aromatic ring. The third first neighbour of the original atom is a C atom in a six-membered aromatic ring with two additional neighbours: a C atom in two six-membered aromatic rings and an H atom. Finally, the third-neighbour composition of the original atom is as follows: an O atom with one bond, two C atoms with four bonds, two H atoms with one bond and four C atoms with three bonds.

Evidently, each atom class encodes its local chemical environment. The number of such atom classes derived from the COD is around 260 000. Since the space of atom classes is very large, if not infinite, it can be expected that some atom types for a new ligand might not be in the list of atom types derived from the COD. One must remember that the purposes of small-molecule and macromolecular crystallography are very different, and thus it can be expected that they have a tendency to target different types of chemical compounds in their studies. Therefore, the probability of a given atom class being absent from the COD, or any other large database of small molecules, is not negligible. Consequently, it is necessary to have some generalization of atom classes. In other words, we need to be able to reduce the information encoded in the atom types in a way that does not lose too much information. The generalization used depends on particular bonds and angles; these are described in the next section.

## 4. Tables of bonds and angles   

Once all atom types have been identified and classified, *AceDRG* creates and organizes tables pertaining to bonds and angles. Since the number of potentially different atom types is infinite, it is possible for a pair of atom types in a given compound, as defined above, to not be in the list of bonds. Therefore, we need well organized tables of atom types, bonds and angles for the fast and efficient searching of exact atom types as well as fast generalization, if and when needed.

The bond tables are organized in a hierarchical manner, with seven levels with various generalizations and fine-tuning. We refer to each level as the ‘generalized’ atom types. These levels are (i) hash code, (ii) combination of hybridization states of atoms, (iii) information about inter-ring and intra-ring bonds, (iv) first-neighbour connections, (v) details about the first-neighbour connections, (vi) atom types without third-neighbour information and (vii) the full atom types. The first level, hash code, encodes basic properties of the individual atom types. The second and third levels contain information about the bonds. The remaining levels comprise properties of the atom types.

The hash codes encode essential chemical properties of the atoms. Each property is defined as an integer number referring to the position of the atom in the property list. These include (i) the position of the element in the periodic table, *i.e.* an integer representation of element names, (ii) the number of connections, (iii) the size of the smallest ring that the atom belongs to and (iv) whether the atom is part of an aromatic ring. If required, other chemical properties can be added. The hash level can already be considered to be a relatively fine-grained atom typing; the number of *AceDRG* hash-level atom types (around 180) is more than that in the *REFMAC* energy library (around 100) for the same extended organic set. One advantage of this hash-code-level atom typing over the current *REFMAC* energy library is that it uses a constructive algorithm, allowing it to be extended easily by adding more chemical information.

### 4.1. Bonds   

The bond tables are organized to facilitate fast searching for atom-type pairs and, if a given pair is not in the table, then to quickly find a reasonable approximation to the atom-type pair, and thus bond lengths. Each line corresponds to a bond record, which comprises the following information about the bonded atom-pair type.

(i) A pair of hash codes.
(ii) The hybridization states for the two bonded atoms. The current hybridization states are *sp* ^1^, *sp* ^2^, *sp* ^3^ and ‘none’.
(iii) An indicator specifying whether this bond is within or between rings.
(iv) The number of neighbours for each of the first neighbours of the atom type under consideration. For example, ‘4:3:3:1’ would indicate that one of the first neighbours has four bonds, two of them have three bonds and one has one bond.
(v) A further elaboration on the previous level, including element names and whether the first neighbours belong to rings. For example, ‘C[5,6]-3_3_3_0:C[5]-3_2_2:H-3:S[6]-3_3’ would indicate that one first neighbour is a C atom that is in a five-membered and a six-membered ring; this C atom is connected to four other atoms. The hybridization states of three of these are *sp* ^3^ and that of one of them is ‘none’ (remembering that H and halogen atoms are assigned a hybridization state of ‘none’ according to *AceDRG* classification).
(vi) Almost-full atom-type information, but without any third-neighbour information.
(vii) Finally, the full atom types for the two bonded atoms, including some third-neighbour information (such as for the atom type C23 described in the previous subsection).

At each level, the average bond length, standard deviation and the number of observations are stored. Note that the number of observations and standard deviations are used in further decision-making.

### 4.2. Searching for bond values in the *AceDRG* tables   

Searching the table (Long *et al.*, 2017 ▸) for a given pair of atom types is performed level by level. If an exact match is found, and the number of observations at this level is more than four, then the corresponding bond length and standard deviation are taken. If not then we repeat the search at a higher level. At each level, we check the number of observations used to calculate the mean bond length and standard deviation. If the standard deviation is more than 0.03, or the number of observations is less than four, then we go to the next level. Otherwise, we accept the mean bond length and standard deviation from this level.

If no candidate entries are found that satisfy these two conditions up to the hash level, we select the lowest level with more than four observations. This applies to all levels where no matching of ‘generalized’ atom types happens. If there is no match of ‘generalized’ atom types, even at the hash level, then we use atom types from the *REFMAC* energy library and use the corresponding simplified bond lengths as fall-back values. In this case, the standard deviation is assigned to be 0.02. It should be noted that in the test of 9000 ligands from the Chemical Component Dictionary (CCD) of the PDB we have not seen a single case where the use of *REFMAC* energy types is necessary.

### 4.3. Angles   

The angle tables are organized similarly to the bond tables, with the one exception that here we use an atom-type triple. Angle-table searches are carried out using a similar algorithm as for the bond tables.

## 5. Ligand description and coordinate generation   

**Figure 5:**
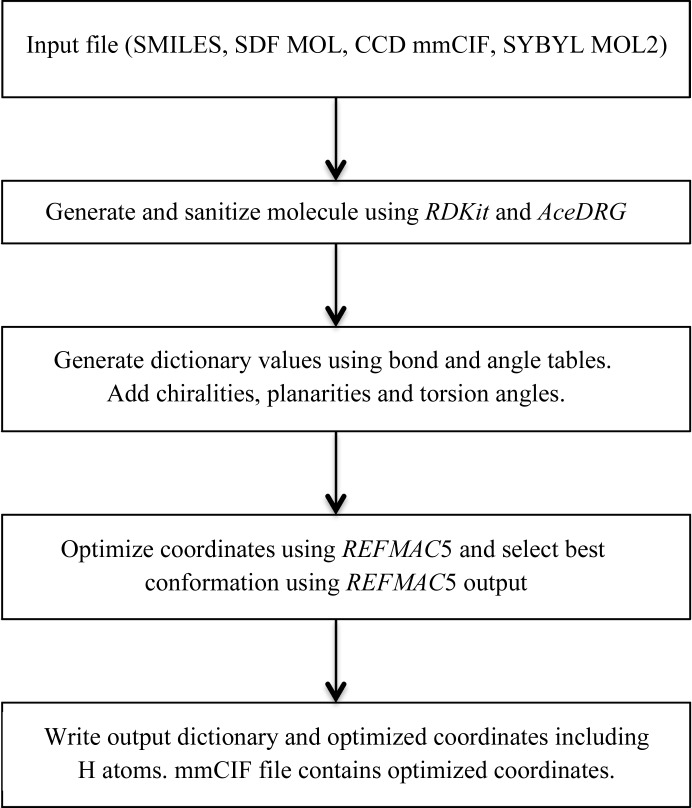
Flow chart of *AceDRG* ligand-description generation.

Fig. 5 ▸ shows a flow chart describing the derivation of stereochemical information and coordinate set(s) using basic chemistry as input. The workflow is relatively simple and comprises four steps.

(i) Read the input file, which contains bonding information. At this step, either *AceDRG* directly (mmCIF format) or *RDKit* is used to read the files and organize minimal information about atoms and bonds. If mmCIF is used as an input file then *AceDRG* checks whether the file contains a SMILES string. If it does, then *RDKit* is used for conformer generation. However, when a SMILES string is used the atom names are lost. *AceDRG* uses an exact graph isomorphism algorithm to match the atom names generated by *RDKit* to those in the input mmCIF file, ensuring that the atom names are retained. If the input mmCIF file does not include a SMILES string then *AceDRG* converts this file to an SDF MOL file (Dalby *et al.*, 1992 ▸) and feeds it to *RDKit* to generate the initial conformation. The current version of *AceDRG* accepts CCD mmCIF, SMILES string, SDF MOL (Dalby *et al.*, 1992 ▸) and SYBYL MOL2 (Clark *et al.*, 1989 ▸) file formats. *RDKit* is used for the interpretation of SMILES, SDF MOL and MOL2 files.
(ii) In the second step, initial models are generated and the molecule is sanitized using both *RDKit* and *AceDRG* functionality. The chemistry of the molecule is verified, ensuring that it conforms to basic chemical rules. In addition, information regarding functional groups and pH is used to proton­ate or deprotonate functional groups such as carboxyl groups, phosphates and sulfate groups. If explicit H atoms are defined in the SMILES string, *AceDRG* retains these H atoms.
(iii) At this stage, atom types are generated for each atom in the initial model. The *AceDRG* tables are then consulted to find the corresponding ‘ideal’ bond and angle values. Plane groups and chiral centres are also added and an initial mmCIF dictionary file is created.
(iv) Finally, the coordinates corresponding to the initial conformations from step (i) are optimized using the idealization mode of *REFMAC*, together with the initial mmCIF dictionary file just generated. The optimized coordinates are then added to the output mmCIF dictionary file. In its default mode, *AceDRG* generates 20 different conformations and then idealizes them before selecting the best one according to *REFMAC*5 geometry information. The final output is an mmCIF dictionary file and a PDB file containing the coordinates.

### 5.1. Chemistry sanitization   

*AceDRG* first uses *RDKit* to sanitize the molecule, making sure that it is consistent with basic chemistry, for example that the numbers of connections and valences are consistent. Then, using functional groups, it assigns formal charges to atoms of groups such as carboxyl, amine, sulfate and phosphate groups. In total there are 25 functional groups used by *AceDRG* at the moment. The number of functional groups can be extended without difficulty.

### 5.2. Planes   

If an atom is in the *sp* ^2^ hybridization state then it, together with all atoms that it is bonded to, are assumed to be on the same plane. If an individual ring is aromatic, all atoms in the ring and their connected outside-ring atoms are in a plane. If a fused multiple-ring system is aromatic then all atoms in each of the smallest rings, together with the atoms that they are bonded to, are considered to be on the same plane. This allows some deformation of large planar systems, such as flavin rings, during refinement if the experimental data are sufficiently strong to indicate that there must be a departure from planarity. However, all atoms of the smallest rings will try to stay on the same plane.

### 5.3. Chiralities   

Just like in a SMILES string, the chiral centres in the monomer library (generated by *AceDRG*) are local chiralities. That is, if the central atom is *sp* ^3^ and the number of bonded non-H atoms is not less than three then the atom is considered to be a chiral centre. If the Cahn–Ingold–Prelog (CIP) priorities of at least two atoms (lone pairs of electrons are considered to be dummy atoms) are the same, or the input file does not have any indication of chiral centres (by coordinates or otherwise), then the sign of the chiral volume is assumed to be ‘both’, indicating that at least two atoms bonding to the central atom can swap places without changing stereochemistry. In some cases, chiral volume signs can be assigned even for nonchiral centres. This can be useful because the atom names in the PDB file make nonchiral centres chiral by nomenclature. If the CIP priorities of atoms bonded to the central atom are different, or the input file indicates that this centre must be chiral with definite sign, then the program considers this centre as a genuine chiral centre with definite sign.

## 6. Examples of application   

Here, we use two examples from the PDB to demonstrate *AceDRG*-generated dictionary values in practice. In general, the bond lengths and angles generated by *AceDRG* seem to be reasonably accurate (Tucker & Steiner, 2017 ▸). The first example aims to demonstrate that although the bond values generated from *AceDRG* are more accurate, and thus the refined structure should in principle be better in terms of chemical structure, the differences between structures refined using different dictionary values are so small that they are barely visible by eye and are unlikely to cause incorrect biological conclusions. The second example demonstrates the importance of aromaticity perception, and how it may affect inferred biological conclusions.

### 6.1. Example 1: PDB entry 3o8h, ligand name O8H (Willand *et al.*, 2010 ▸)   

**Figure 6:**
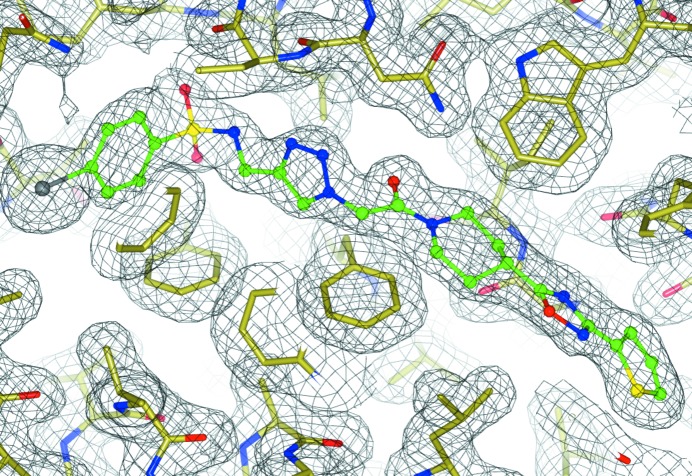
An example of a ligand refined using *AceDRG* restraints: PDB entry 2o8h with the ligand O8H (Willand *et al.*, 2010 ▸). Although the bond lengths are significantly different, the overlaid structures are visually almost identical. This figure was produced using *CCP*4*mg* (McNicholas *et al.*, 2011 ▸).

The electron density corresponding to the ligand (Fig. 6 ▸) is of sufficient quality, with the exception of the iodinated benzene ring (this might be owing to radiation damage resulting in partial cleavage of the I atom, causing slight disorder of the benzene ring). Fig. 6 ▸ demonstrates that *AceDRG* perceives the aromaticity of the rings correctly. The bond distance between N21 and N22 in the PDB file is around 1.22 Å, which is shorter than it should be. The corresponding *AceDRG*-derived bond length is around 1.32 Å (for the full dictionary, see Supporting Information), which seems to reflect the fact that this ring is aromatic and the bond length is longer than a double bond (around 1.24 Å) but shorter than a single bond (around 1.41 Å). Unfortunately, with current PDB entries it is impossible to compare *AceDRG*-derived dictionary values (or values produced by any other software) with those used during the analysis of the PDB structures. Nevertheless, despite the apparently large differences between the bond lengths, the overlaid ligands before and after refinement with *AceDRG* dictionary values show very little visible difference.

### 6.2. Example 2: FAD *versus* FADH_2_, PDB entry 3hdy, ligand name FDA   

As is well known, FAD/FADH_2_ conversion plays an important role in many biological processes. However, there seems to be a great deal of confusion in labelling and refining this cofactor. One of the problems is that in many calculations the flavin group is assumed to be a flat plane. However, even in FAD the flavin can be bent, although not as much as in FADH_2_. There is also a half-oxidized state of the flavin moiety that is usually not considered in detail. The reason for this is that the half-oxidized state is an intermediate between the fully reduced and fully oxidized states, and the probability of observing this state in isolation is very small. However, if the structural environment is favourable then the half-oxidized state could be stabilized. In general, while testing various ligands we came to the conclusion that there needs to be some initiative similar to *PDB_REDO* (Joosten *et al.*, 2012 ▸) to reanalyse all ligands in the PDB. The most challenging part of such a project would be the analysis of the stability of compounds in isolation and in the structural environments that they are in. FAD is one of the examples that requires special attention.

**Figure 7:**
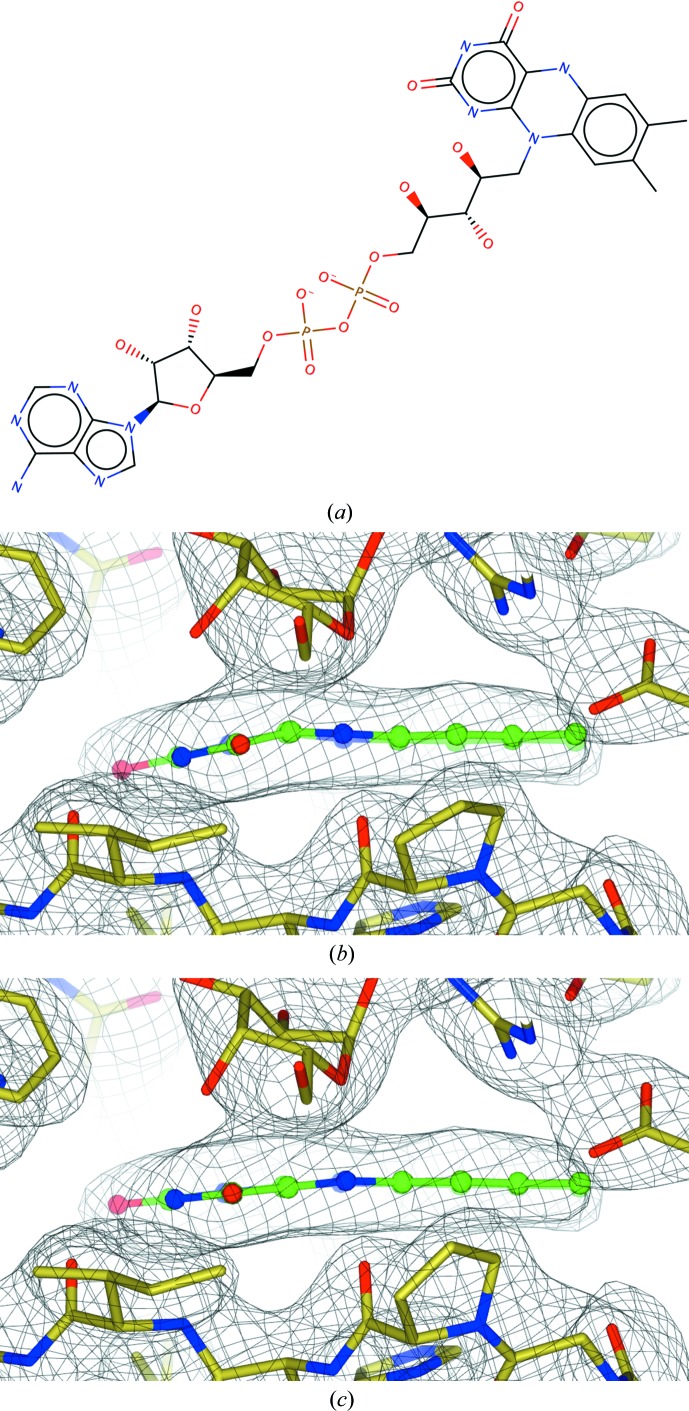
Dictionary and refinement of FADH_2_: PDB entry 3hdy with the ligand FDA (Partha *et al.*, 2009 ▸). (*a*) The FADH_2_ cofactor. *AceDRG* perceives aromatic rings and the ring system. Phosphate groups have a −1 charge. (*b*) The flavin ring in the electron density before refinement using the *AceDRG* dictionary; (*c*) the flavin ring in the electron density after refinement using the *AceDRG* dictionary. (*a*) was produced using *Marvin Sketch* v.16.9.12 (http://www.chemaxon.com) and (*b*) and (*c*) were produced using *CCP*4*mg* (McNicholas *et al.*, 2011 ▸).

PDB entry 3hdy (Partha *et al.*, 2009 ▸) contains several ligands, including FAD and FDA, representing FAD and FADH_2_. Our focus is only on FDA. Fig. 7 ▸ shows the geometry and the electron density before and after refinement using *AceDRG* dictionary values (for the full dictionary, see Supporting Information). It is evident that after refinement the flavin plane becomes flatter, and deformation of the plane is smooth over the whole flavin moiety. Analysis of the electron density and ligand alone cannot give a definite answer about the oxidation state of the ligand; one would need to use other complementary techniques for this. However, electron density and ligand geometry, if handled with care, can become crucial pieces of evidence suggesting favourability of one or another state.

## 7. Conclusions and future perspectives   

The program *AceDRG* has been designed to extract and organize atom types from small-molecule databases. The current version uses the freely available COD, although the algorithms and implementations are flexible, and any source of reliable small-molecule coordinate sets can be used to supplement/update/replace the relevant tables.

Tests show that *AceDRG* works reasonably well for a large class of cases without metals. However, there are still problems with some of the cases. One case to note is N with three connections where one of the atoms it is bonded to is *sp* ^2^. Owing to similarities in electronic structures, we expect B to exhibit a similar type of behaviour. By default these atoms are considered to be *sp* ^2^, with some correction added in order to account for the local environment. In many cases, we can make decisions regarding the hybridization states of N using the local environment. However, there are a number of cases where it is hard, if possible at all, to make such decisions. One can imagine cases where the same type of N atom with similar covalent environments might have *sp* ^2^ or *sp* ^3^ hybridization depending on their environment. The N atom within the piperidine group is one such example. If this N atom is bound to an *sp* ^2^ C atom then it can be in the *sp* ^2^ or *sp* ^3^ state. Moreover, there may be cases where the hybridization state of N could be an intermediate between *sp* ^2^ and *sp* ^3^. To deal with such cases, small-molecule databases such as the COD would need to be analysed and such cases identified. Subsequently, the hybridization state and thus the geometric parameters of the whole compound would need to be adjusted depending on the environment.

Metal-containing compounds pose special problems. There are a multitude of problems that need to be contemplated carefully and dealt with robustly before we can claim that we can deal with metal-containing compounds for MX and cryo-EM fitting.

(i) The coordination and local geometry of some of the metals depend on their oxidation states. During data collection it cannot be guaranteed that the oxidation state of the metal will stay unchanged.
(ii) Metal-containing ligands cannot always be considered independently from their protein environment. For example, any of the six ligands of Mg^2+^ can be replaced with some of the atoms from proteins. Therefore, metal-containing compounds may require another level of abstraction, where the surrounding atoms can be exchanged with other atoms without affecting local geometry. Some work towards context-dependent metal-coordination behaviour has been discussed by Touw *et al.* (2016 ▸).
(iii) The same metal may exist with different coordination geometry.
(iv) Whilst in many cases metals are very well visible in electron density produced by X-ray crystallography, their coordinating ligands may be invisible owing to the series-termination effect being amplified around metals owing to their high electron density. Future approaches will need to be able to predict missing coordinating atoms, which might be a tricky problem to approach as there may not be unique coordination geometry for a given metal. One promising approach is the bond-valence theory advocated by Brown (2009 ▸). This approach should not be confused with the well known valence-bond theory of molecular orbitals. To apply this approach we need to analyse the whole COD, classify metals with their environments and then apply them to coordinates as necessary. This approach has been successfully applied (Zheng *et al.*, 2017 ▸).

By default the current version of *AceDRG* presumes that the pH of the environment of the compound is 7.0, but this can be overridden by the user. This approach covers a sufficiently large class of problems. However, one can imagine cases where the local environment of a ligand is different and the same ligand can exist with different protonation states in different environments. If there are only one or two proton­ation states then such cases can be tabulated and the decision as to which ligand geometry definition to use can be made during model building and refinement. However, if the number of protonation states is very large then a better approach could be interactively changing the protonation states of particular regions of a ligand during model building. This would require interaction between model-building programs (*e.g. Coot*) and ligand description-generator programs (*e.g. AceDRG*). This may allow sufficient flexibility, although it requires the user to have sufficient knowledge about chemistry. If such an approach is to be used then the programs should be able to guide users by suggesting the best possible protonation states of particular regions in a particular environment.

*AceDRG* assumes that each tautomer is one independent ligand. If the number of different tautomerization states is small then it would be possible to generate descriptions of all tautomers and use them as and when they are needed. However, if the number of such states is very large then the interaction between *Coot* and *AceDRG* must be designed so as to decide the best possible tautomers depending on the environment. Again, there must be certain chemical intelligence in the model-building program (*Coot*) in order to suggest the best tautomerization state consistent with the current environment of the ligand.

One of the problems that has not been dealt with here is the position of H atoms. It is unlikely that positions of the H atoms in the models from the COD (and from the CSD) have sufficient accuracy, unless experiments are based on neutron diffraction. In many cases H atoms are added in their riding positions (Sheldrick, 2008 ▸), and thus H atoms in the COD (and the CSD) are unlikely to reflect the observations alone; they reflect the prior knowledge regarding chemistry used by the programs generating it, and only to some degree the experimental data. Even if H atoms have been refined using experimental data alone, it is unlikely that their positions can be considered to be particularly accurate; if neutron diffraction is used then H-atom positions will reflect the positions of protons. If X-ray diffraction is used then H atoms should reflect the positions of electrons; even at very high resolution these positions are much less accurate than those of heavier atoms. In general, we need to consider X-ray, neutron and electron diffraction experiments: X-rays see electrons, neutrons see nuclei positions and electrons see both. Thus, in future updates of the dictionary of monomers we will need to consider all of these cases. Perhaps we will need to carry out high-level QM calculations for a small set of molecules in order to derive proton and electron positions for various atom types. Even this will not be a complete solution for the hydrogen problems: the position and electron density around H atoms may depend on their environments.

*AceDRG* is a standalone program, distributed by CCP4, which can be used *via* the command line or embedded within a graphical user interface. Currently it does not have its own GUI. In future, programs such as *JLigand* (Lebedev *et al.*, 2012 ▸) and *Lidia* (Emsley *et al.*, 2010 ▸) will need to be adapted to make the program accessible to wider range of users.

## Supplementary Material

AceDRG description of the ligand O8H. DOI: 10.1107/S2059798317000067/ba5260sup1.txt

AceDRG description of FDA (FADH2). DOI: 10.1107/S2059798317000067/ba5260sup2.txt

## Figures and Tables

**Table 1:** Default hybridization states for atoms The hybridization states of H and halogen atoms are considered to be ‘none’ so that they do not affect decisions regarding the hybridization states of other atoms.

Element	C	N/B	O	S/SE	P
Hybridization states	Connections: 4	Connections: 4	Connections: 2	Connections: 4	Connections: 4
	*sp* ^3^	*sp* ^3^	*sp* ^3^	*sp* ^3^	*sp* ^3^
				
	Connections: 3	Connections: 3	Connections: 1	Connections: 3	Connections: 3
	*sp* ^2^	*sp* ^3^	*sp* ^2^	*sp* ^3^	*sp* ^2^
				
	Connections: 2	Connections: 2		Connections: 2	
	*sp* ^1^	*sp* ^2^		*sp* ^3^	

**Table 2:** The number of π electrons contributed by each atom in all *sp* ^2^ ring systems Note: all atoms in a ring must be in the *sp* ^2^ hybridization state; otherwise the ring is not aromatic and π electrons are not counted.

	Elements					
No. of π electrons contributed	C	N	O	S	P	B
2	Connection: 3	Connection: 3	Connection: 2	Connection: 2	Connection: 3	
	Charge: −1	Charge: 0	Charge: 0	Charge: 0	Charge: 0	
					
		Connection: 2				
		Charge: −1				
					
1	Connection: 3	Connection: 3	Connection: 2	Connection: 2	Connection: 3	Connection: 3
	Charge: 0	Charge: 1	Charge: 1	Charge: 1	Charge: 1	Charge: −1
					
	Connection: 2	Connection: 2		Connection: 3	Connection: 2	
	Charge: −1	Charge: 0		Charge: 0	Charge: 0	
					
0	Connection: 3	Connection: 2				Connection: 3
	Charge: 1	Charge: 1				Charge: 0
					
	Connection: 3 (double to an outside-ring atom, *e.g.* O)					
	Charge: 0					
					
	Connection: 2					
	Charge: 0					

## References

[bb1] Adams, P. D. *et al.* (2010). *Acta Cryst.* D**66**, 213–221.

[bb2] Balaban, A. T. (1985). *J. Chem. Inf. Model.* **25**, 334–343.

[bb3] Berman, H. M. *et al.* (2002). *Acta Cryst.* D**58**, 899–907. 10.1107/s0907444902003451 12037327

[bb4] Brown, I. D. (2009). *Chem. Rev.* **109**, 6858–6919. 10.1021/cr900053k PMC2791485 19728716

[bb5] Bruno, I. J., Cole, J. C., Kessler, M., Luo, J., Motherwell, W. D. S., Purkis, L. H., Smith, B. R., Taylor, R., Cooper, R. I., Harris, S. E. & Orpen, A. G. (2004). *J. Chem. Inf. Comput. Sci.* **44**, 2133–2144. 10.1021/ci049780b 15554684

[bb7] Chivers, T. (2005). *A Guide to Chalcogen–Nitrogen Chemistry.* Singapore: World Scientific.

[bb8] Chivers, T. & Manners, I. (2009). *Inorganic Rings and Polymers of the p-Block Elements: From Fundamentals to Applications*. London: Royal Society of Chemistry.

[bb9] Clark, M., Cramer, R. D. & Van Opdenbosch, N. (1989). *J. Comput. Chem.* **10**, 982–1012.

[bb10] Coulson, C. A., O’Leary, B. & Mallion, R. B. (1978). *Hückel Theory for Organic Chemists*. London: Academic Press.

[bb11] Dalby, A., Nourse, J. G., Hounshell, W. D., Gushurst, A. K. I., Grier, D. L., Leland, B. A. & Laufer, J. (1992). *J. Chem. Inf. Model.* **32**, 244–255.

[bb12] Dimitropoulos, D., Ionides, J. & Henrick, K. (2006). *Curr. Protoc. Bioinformatics*, Unit 14.3. https://doi.org/10.1002/0471250953.bi1403s15. 10.1002/0471250953.bi1403s15 18428761

[bb13] Downs, G. M., Gillet, V. J., Holliday, J. D. & Lynch, M. F. (1989). *J. Chem. Inf. Model.* **29**, 172–187.

[bb14] Emsley, P., Lohkamp, B., Scott, W. G. & Cowtan, K. (2010). *Acta Cryst.* D**66**, 486–501. 10.1107/S0907444910007493 PMC2852313 20383002

[bb15] Engh, R. A. & Huber, R. (1991). *Acta Cryst.* A**47**, 392–400.

[bb16] Engh, R. A. & Huber, R. (2001). *International Tables for Crystallo­graphy*, Vol. *F*, edited by M. G. Rossmann & E. Arnold, pp. 382–392. Dordrecht: Kluwer Academic Publishers.

[bb17] Feng, G., Chen, L., Maddula, L., Akcan, O., Oughtred, R., Berman, H. M. & Westbrook, J. (2004). *Bioinformatics*, **20**, 2153–2155. 10.1093/bioinformatics/bth214 15059838

[bb18] Figueras, J. (1996). *J. Chem. Inf. Comput. Sci.* **36**, 986–991.

[bb19] Fowler, P. W., Rees, C. W. & Soncini, A. (2004). *J. Am. Chem. Soc.* **126**, 11202–11212. 10.1021/ja046399z 15355101

[bb20] Gražulis, S., Chateigner, D., Downs, R. T., Yokochi, A. F. T., Quirós, M., Lutterotti, L., Manakova, E., Butkus, J., Moeck, P. & Le Bail, A. (2009). *J. Appl. Cryst.* **42**, 726–729. 10.1107/S0021889809016690 PMC3253730 22477773

[bb21] Gražulis, S., Daškevič, A., Merkys, A., Chateigner, D., Lutterotti, L., Quirós, M., Serebryanaya, N. R., Moeck, P., Downs, R. T. & Le Bail, A. (2012). *Nucleic Acids Res.* **40**, D420–D427. 10.1093/nar/gkr900 PMC3245043 22070882

[bb22] Groom, C. R., Bruno, I. J., Lightfoot, M. P. & Ward, S. C. (2016). *Acta Cryst.* B**72**, 171–179. 10.1107/S2052520616003954 PMC4822653 27048719

[bb23] Hanser, T., Jauffret, P. & Kaufmann, G. A. (1996). *J. Chem. Inf. Model.* **36**, 1146–1152.

[bb24] Joosten, R. P., Joosten, K., Murshudov, G. N. & Perrakis, A. (2012). *Acta Cryst.* D**68**, 484–496. 10.1107/S0907444911054515 PMC3322608 22505269

[bb25] Karunan Partha, S., van Straaten, K. E. & Sanders, D. A. (2009). *J. Mol. Biol.* **394**, 864–877. 10.1016/j.jmb.2009.10.013 19836401

[bb26] Krygowski, T. M., Cyrański, M. K. & Matos, M. A. R. (2009). *Aromaticity in Heterocyclic Compounds*. Berlin, Heidelberg: Springer-Verlag.

[bb27] Kühlbrandt, W. (2014). *Elife*, **3**, e03678. 10.7554/eLife.03678 PMC4131193 25122623

[bb28] Leach, A. R., Dolata, D. P. & Prout, K. (1990). *J. Chem. Inf. Model.* **30**, 316–324. 10.1021/ci00067a017 2211887

[bb29] Lebedev, A. A., Young, P., Isupov, M. N., Moroz, O. V., Vagin, A. A. & Murshudov, G. N. (2012). *Acta Cryst.* D**68**, 431–440. 10.1107/S090744491200251X PMC3322602 22505263

[bb30] Liebeschuetz, J., Hennemann, J., Olsson, T. & Groom, C. R. (2012). *J. Comput. Aided Mol. Des.* **26**, 169–183. 10.1007/s10822-011-9538-6 PMC3292722 22246295

[bb31] Long, F., Nicholls, R. A., Emsley, P., Gražulis, S., Merkys, A., Vaitkus, A. & Murshudov, G. N. (2017). *Acta Cryst.* D**73**, 103–111. 10.1107/S2059798317000079 PMC5297913 28177306

[bb32] McNicholas, S., Potterton, E., Wilson, K. S. & Noble, M. E. M. (2011). *Acta Cryst.* D**67**, 386–394. 10.1107/S0907444911007281 PMC3069754 21460457

[bb33] Moriarty, N. W., Grosse-Kunstleve, R. W. & Adams, P. D. (2009). *Acta Cryst.* D**65**, 1074–1080. 10.1107/S0907444909029436 PMC2748967 19770504

[bb34] Murshudov, G. N., Skubák, P., Lebedev, A. A., Pannu, N. S., Steiner, R. A., Nicholls, R. A., Winn, M. D., Long, F. & Vagin, A. A. (2011). *Acta Cryst.* D**67**, 355–367. 10.1107/S0907444911001314 PMC3069751 21460454

[bb35] Nicholls, R. A., Long, F. & Murshudov, G. N. (2012). *Acta Cryst.* D**68**, 404–417. 10.1107/S090744491105606X PMC3322599 22505260

[bb36] Parkinson, G., Vojtechovsky, J., Clowney, L., Brünger, A. T. & Berman, H. M. (1996). *Acta Cryst.* D**52**, 57–64. 10.1107/S0907444995011115 15299726

[bb37] Pozharski, E., Weichenberger, C. X. & Rupp, B. (2013). *Acta Cryst.* D**69**, 150–167. 10.1107/S0907444912044423 23385452

[bb39] Reynolds, C. H. (2014). *ACS Med. Chem. Lett.* **5**, 727–729. 10.1021/ml500220a PMC4094245 25050154

[bb40] Rocha, G. B., Freire, R. I., Simas, A. M. & Stewart, J. J. P. (2006). *J. Comput. Chem.* **27**, 1101–1111. 10.1002/jcc.20425 16691568

[bb41] Schröder, G. F., Levitt, M. & Brunger, A. T. (2010). *Nature (London)*, **464**, 1218–1222. 10.1038/nature08892 PMC2859093 20376006

[bb42] Schüttelkopf, A. W. & van Aalten, D. M. F. (2004). *Acta Cryst.* D**60**, 1355–1363. 10.1107/S0907444904011679 15272157

[bb43] Sheldrick, G. M. (2008). *Acta Cryst.* A**64**, 112–122. 10.1107/S0108767307043930 18156677

[bb44] Smart, O. S., Womack, T. O., Flensburg, C., Keller, P., Paciorek, W., Sharff, A., Vonrhein, C. & Bricogne, G. (2012). *Acta Cryst.* D**68**, 368–380. 10.1107/S0907444911056058 PMC3322596 22505257

[bb45] Smart, O. S., Womack, T. O., Sharff, A., Flensburg, C., Keller, P., Paciorek, W., Vonrhein, C. & Bricogne, G. (2011). *Grade* v.1.1.1. Global Phasing Ltd, Cambridge, England.

[bb48] Steiner, R. & Tucker, J. (2017). *Acta Cryst.* D**73**, 93–102. 10.1107/S2059798316017964 PMC5297912 28177305

[bb46] Szabo, A. & Ostlund, N. S. (1989). *Modern Quantum Chemistry: Introduction to Advanced Electronic Structure Theory*. New York: McGraw–Hill.

[bb47] Touw, W. G., van Beusekom, B., Evers, J. M. G., Vriend, G. & Joosten, R. P. (2016). *Acta Cryst.* D**72**, 1110–1118. 10.1107/S2059798316013036 PMC5053137 27710932

[bb49] Vagin, A. A., Steiner, R. A., Lebedev, A. A., Potterton, L., McNicholas, S., Long, F. & Murshudov, G. N. (2004). *Acta Cryst.* D**60**, 2184–2195. 10.1107/S0907444904023510 15572771

[bb50] Walsh, J. D. & Miller, A.-F. (2003). *J. Mol. Struct.* **623**, 185–195.

[bb51] Weichenberger, C. X., Pozharski, E. & Rupp, B. (2013). *Acta Cryst.* F**69**, 195–200. 10.1107/S1744309112044387 PMC3564628 23385767

[bb52] Weininger, D. (1988). *J. Chem. Inf. Model.* **28**, 31–36.

[bb53] Weininger, D., Weininger, A. & Weininger, J. L. (1989). *J. Chem. Inf. Model.* **29**, 97–101.

[bb54] Wilberg, E., Wilberg, N. & Holleman, A. F. (2001). *Inorganic Chemistry.* New York: Academic Press.

[bb55] Willand, N., Desroses, M., Toto, P., Dirié, B., Lens, Z., Villeret, V., Rucktooa, P., Locht, C., Baulard, A. & Deprez, B. (2010). *ACS Chem. Biol.* **5**, 1007–1013. 10.1021/cb100177g 20704273

[bb56] Winn, M. D. *et al.* (2011). *Acta Cryst.* D**67**, 235–242.

[bb57] Zheng, H., Langner, K. M., Shields, G. P., Hou, J., Kowiel, M., Allen, F. H., Murshudov, G. N. & Minor, W. (2017). *Acta Cryst.* D**73**, https://doi.org/10.1107/S2059798317000584. 10.1107/S2059798317000584 PMC5503122 28375143

